# Spinal articular dysfunction is common in athletic horses

**DOI:** 10.1111/evj.14541

**Published:** 2025-06-01

**Authors:** Claudia R. Patricio, Gustavo H. Z. Winter, Petra Garbade

**Affiliations:** ^1^ Veterinary Faculty Federal University of Rio Grande do Sul Porto Alegre Brazil; ^2^ International Veterinary Chiropractic Association (IVCA) Committee Member Hamburg Germany

**Keywords:** back pain, horse, spinal dysfunction, veterinary chiropractic

## Abstract

**Background:**

Spinal articular dysfunction disrupts normal neural function and may lead to stiffness, pain or poor performance. Back pain is common in equine athletes, and it is a common complaint by horse owners requesting chiropractic evaluations.

**Objectives:**

To describe the prevalence of spinal articular dysfunction in showjumping horses.

**Study Design:**

Retrospective analysis of clinical records.

**Methods:**

Records from 3 years of examinations in horses, from beginners to high‐performance levels. Each horse had 30 spinal segments evaluated for the presence of dysfunctional articulations. Exams were performed by a certified International Veterinary Chiropractic Association veterinarian. Data recorded included age, sex, complaint and history of previous veterinary chiropractic examinations. Descriptive and statistical analysis was performed by age groups: G1 up to 6 years old, G2 from 7 to 10 years, G3 from 11 to 14 years, and G4 from 15 years and above. Significance was set as *p* < 0.05; results were presented as mean ± SD.

**Results:**

Four hundred and ninety‐two jumping horses were included (4–19 years old). On average, animals had 11.9 (±3.5) dysfunctional spinal segments. The minimal number observed was 2 and the maximum was 23. No animals were free of spinal dysfunction, and 442 (89.8%) were found to have segmental dysfunction in all 3 spinal regions. The most affected vertebrae were L3, L2, L4, C1 and T7. Dysfunctional segments in the cervical region were diagnosed less frequently in G1 horses (*p* < 0.05).

**Main Limitations:**

Objective pain measurements were unavailable. Lameness, when present, was not graded and was not directly correlated with spinal dysfunction.

**Conclusions:**

Jumping horses had a high prevalence of spinal articular dysfunction, mainly in the lumbar spine, thoracic sling area and atlanto‐occipital joint. The prevalence of dysfunction of the cervical spine increases with age. No equine athlete in this study was free of spinal articular dysfunction.

## 1 INTRODUCTION

Common complaints leading to requests for chiropractic evaluation of horses include back pain, asymmetrical movement and reduced suppleness.^1^, ^2^ Back pain is particularly common in equine athletes due to the significant physical and physiological stress associated with their activities^3^ and the prevalence of back problems in horses varies greatly (from 0.9% to 94%), depending on the specialisation or type of practice surveyed; that is, general practice 0.9%; veterinary school referrals 5%; mixed equine practice (dressage, jumpers and eventing) 13%; spinal research clinic 47% or equine chiropractic clinic 94%.^4^

According to chiropractic theory, spinal articular dysfunction, also referred to as the vertebral subluxation complex (VSC), disrupts normal neural function and is characterised by spinal segments with hypomobile facet joints and impaired neural and connective tissue function.^4^ Restoring normal movement and function of the spine is believed to enhance neural function and support musculoskeletal health.^1^, ^4^ Previous studies evaluating spinal segmental dysfunctions demonstrated a relation with sportive disciplines and back pain,^3^, ^5^, ^6^, ^7^ but there is a lack of information regarding showjumping horses with a large and well‐defined population regarding their age and competitive levels.

The aim of this retrospective study was to identify the spinal regions most susceptible to spinal articular dysfunctions in jumping horses using the data from the veterinary chiropractic records of an experienced DVM, certified by the International Veterinary Chiropractic Association. It was anticipated that there are specific spinal regions, such as the thoracolumbar junction and cervical spine, that are more susceptible to articular dysfunctions due to the biomechanical stresses associated with jumping activities, and this would be more prevalent in older athletes.

## 2 MATERIALS AND METHODS

### 2.1 Database collection

Records from a total of 492 jumping horses, predominantly Warmbloods, were reviewed. Inclusion criteria were records with available data on horse identification, age, sex, presenting complaint, history of previous examination, and horses that had all 30 spinal segments evaluated. Additional data from back pain and/or lameness were included in the analysis when present. Records that failed to provide this minimum data and records from subsequent examinations of the same horse were not included. Also, files that described any previous chiropractic manipulation within weeks prior to the exam were excluded to minimise potential carryover effects from previous treatments.

The horses had not been subjected to any prior specific rest or work routines. The assessments were performed for routine/preventive care examinations or for the treatment of complaints commonly including lack of flexibility, back pain, decreased performance or lameness. When available, information on lameness and back pain was recorded, but no further examination regarding the grading of lameness was performed.

The database was collected from routine veterinary chiropractic practice and spanned 3 years of records. Information was recorded in individual files detailing the location and frequency of spinal dysfunctions for all 492 subjects. Age data were available for 177 animals, muscle tone data for 470 patients and signs of back pain for 467 animals.

### 2.2 Animals

The cohort comprised 207 geldings, 249 mares and 39 stallions, with ages ranging from 4 to 19 years (mean age 9.9 years). These horses participated in diverse jumping classes, with fence heights ranging from 80 cm to 1.60 m, and were ridden by beginner‐level to professional riders. The subjects were sourced from 11 distinct riding clubs located in Southern Brazil, specifically in the states of Rio Grande do Sul, Santa Catarina, Paraná and São Paulo. For analytical purposes, the horses were categorised by age and/or training level as follows:

Group 1: Up to 6 years old; beginning and developing training abilities.
Group 2: Ages 7–10 years; consolidation of performance phase.
Group 3: Ages 11–14 years; peak performance phase.
Group 4: Ages 15 years and above; a phase where many showjumpers begin to experience a decline in athletic performance.

### 2.3 Veterinary chiropractic examination

The chiropractic examination was conducted by a doctor of veterinary medicine (DVM) certified by the International Veterinary Chiropractic Association (IVCA). Evaluations were requested by the horses' primary clinician or by their owners, riders and trainers. The number of prior treatments varied among the horses; some had never received veterinary chiropractic care before this examination, while others had undergone previous regular treatment. The horses were examined while at rest on level ground, either held by hand or tied up with a halter. Muscle tone and signs of muscle pain were assessed subjectively based on clinical signs and behavioural indicators.

Use of slow and gradual direct manual palpation of the cervical, thoracolumbar and gluteal musculature was used to assess muscle tone, presence of spasms or fasciculations and signs of muscular pain. Behavioural indicators such as tail swishing, sudden changes in posture, pinned ears, attempts to kick or bite, or repeated pulling away during the examination were recorded as clinical pain reactions. In cases where pain signs were ambiguous, palpation was repeated and only noted if the response was consistently the same. Muscle tone was classified upon examiner experience based on its elastic and contractile properties. Muscles that felt elastic and absent from contractile activities upon palpation were classified as having good tone. Muscles that felt rigid were noted for increased tone. Hypertonic bands or nodules within the muscle were interpreted as muscle contractions and were generally painful to palpation. Contractile muscle activities upon palpation were interpreted as discomfort or pain.

Dysfunctional spinal segments were diagnosed through direct level‐by‐level motion palpation and assessment of facet joint range of motion along the vertebral spine. Data collected included only the location of dysfunctional segments—excluding direction and degree—to simplify the location profile of them. For the study, each named vertebra corresponded to the cranial motor unit, that is, when L3 is listed it refers to the articulation of L2/L3.

The horse's vertebral column was assessed for a total of 30 vertebral motion units: 7 cervical, 16 thoracic, 6 lumbar and 1 sacral unit. The study included segments from C1 (atlanto‐occipital joint) to C7 in the cervical spine; T3 to T18 in the thoracic spine; L1 to L6 in the lumbar spine; and the sacrum assumed as a single motion unit. Notably, T1 and T2 were excluded due to their anatomical positioning behind the scapula wich made it unassessable.

### 2.4 Data analysis

Normality of data distribution were first assessed with the Shapiro–Wilk test. Further descriptive statistical analysis regarding frequencies and locations of dysfunctional spinal segments was run with ANOVA, Tukey's multiple comparison test, confidence interval (CI) determination. Fisher's exact tests were used to determine the association between age groups. Tests were performed by age groups: G1 up to 6 years old, G2 from 7 to 10 years, G3 from 11 to 14 years and G4 from 15 years and above. Descriptive analysis was performed in Excel (Microsoft Corporation); SPSS software (IBM Analytics) was used for statistical analysis. Since the data were normally distributed, they were reported as mean ± SD, and a *p* value <0.05 was considered significant.

## 3 RESULTS

The horses in this study had 11.9 (±3.5) dysfunctional segments along their spine, representing 39% of the segments evaluated. The minimal number of dysfunctional segments observed per patient was 2 (6% of spine segments) and the maximum was 23 (76% of spine). The mean number of dysfunctional segments for the cervical spine was 2.7 (±1.4); for the thoracic spine 5.8 (±2.8) and 2.6 (±1.3) for the lumbar spine, representing respectively 38%, 35% and 43% of each region. The sacrum (here considered the lumbosacral junction, L6/S1) presented dysfunction in 199 animals, representing 24.4% of examined horses.

A total of 442 subjects (89.8%) had segmental dysfunction in all 3 spinal regions, 47 (9.5%) prevalence in 2 spinal regions and only 3 animals (0.6%) prevalence with a single spinal region affected (sacrum was excluded from this analysis). Seventeen subjects were free of cervical segmental dysfunction and no horse had all 7 cervical segments affected. Seven animals were free of thoracic segmental dysfunction and only one had all 16 segments affected. Twenty‐nine animals had no lumbar segmental dysfunction and 23 had all 6 segments affected. No horse (0/492) was free of spinal dysfunction in this study.

The motion units affected in over 50% of cases were L3 (85.7%), L2 (80.3%), L4 (46.4%), C1 (59.8%) and T7 (51.1%). Table 1 describes the total number of observations and the location of dysfunctional spinal segments, and the percentage in which each vertebra was affected in relation to its spinal segment.

**TABLE 1:** Frequency and percentage of dysfunctional segments (VSC) found at cervical, thoracic, lumbar spinal segments and the sacrum from 492 showjumping horses, descriptive study.

Spinal segment		VSC frequency (*n*)	Percentage of cases (%)
Cervical	C1	284	59.8
	C2	148	31.2
	C3	139	29.3
	C4	193	40.6
	C5	215	45.3
	C6	178	37.5
	C7	159	33.5
Thoracic	T3	108	22.3
	T4	159	32.8
	T5	201	41.4
	T6	226	46.6
	T7	248	51.1
	T8	231	47.6
	T9	199	41.0
	T10	205	42.3
	T11	174	35.9
	T12	147	30.3
	T13	165	34.0
	T14	164	33.8
	T15	150	30.9
	T16	119	24.5
	T17	136	28.0
	T18	179	36.9
Lumbar	L1	294	63.5
	L2	372	80.3
	L3	397	85.7
	L4	298	64.4
	L5	145	31.3
	L6	71	15.3
Sacrum	Sacrum	199	24.4

*Note*: VSC = vertebral subluxation complex, same as dysfunctional spinal segments.

No significant difference in the mean number of affected vertebrae (*p* > 0.05) was found between age groups for thoracic, lumbar and sacral regions. However, there was a significant difference in the mean number of dysfunctional segments in the cervical region (*p* < 0.05) between Groups 1 and 4 (Table 2).

**TABLE 2:** Frequency and mean number of VSC at cervical spine per age group from 177 showjumping horses.

Age group (*n*)	Mean (SD)	95% confidence interval		Minimum VSC	Maximum VSC
		Lower limit	Upper limit		
1 (24)	2.1 (±1.0)^a^	1.7	2.5	0	5
2 (83)	2.4 (±1.4)^ab^	2.1	2.7	0	6
3 (44)	2.9 (±1.3)^b^	2.5	3.7	0	6
4 (26)	2.9 (±1.1)^b^	2.5	3.4	1	6

*Note*: (a, b) different superscripts at column differ, *p* < 0.05. VSC = vertebral subluxation complex, same as dysfunctional spinal segments.

Group 1 had significantly fewer cervical dysfunctional segments (*p* < 0.05) than Groups 3 and 4. The mean number of cervical dysfunctional segments for the younger group was 2.1 (95% CI, 1.7–2.5) and for the older groups was 2.9 (95% CI, 2.5–3.4). There was no significant difference between the mean number of spinal segmental dysfunction observed between sexes (*p* > 0.05).

The patients were submitted to direct muscle palpation to subjectively identify the presence or absence of pain and alterations in muscle tone. Of the 467 subjects where data about signs of back pain was available, 295 (63.2%) had no signs of pain, 88 (18.8%) had discomfort presenting with muscle spasm and fasciculations, and 84 (18%) had clinical signs of muscle pain. From the 470 horses with data about muscle tone (cervical, thoracolumbar and gluteal area), 177 of them (37.7%) had good muscle tone, 252 (53.6%) had increased muscle tone, and 41 (8.7%) had muscle contractures, characterised by shortening and stiffness of muscle tissue. No horse had a note of a decrease in muscle tone in this study.

## 4 DISCUSSION

The most striking finding of this retrospective study was the absence of athletes without segmental spinal dysfunction. Every horse exhibited at least two dysfunctional spinal segments. Furthermore, nearly 90% of the examined horses (442/492) had dysfunction in all spinal regions, excluding the sacrum. Notably, only 3 horses in this study had only a single spinal region affected. This suggests that, regardless of performance levels and the varying skill of riders, every horse exhibited a dysfunctional spinal segment that could potentially impact athletic performance.

A previous study reported that a significant percentage (74%) of horses were severely affected, with a mean of 25% (±19.3%) of dysfunctional spinal segments across all groups,^3^ which is considerably lower than the 39.8% found in the present study. Although 74% of subjects were classified as severely affected, only 60% had multiple spinal regions impacted. In contrast, nearly all horses in the current study (99.4%) exhibited dysfunctions in more than one spinal region. Additionally, the previous study did not specify the number of spinal segments assessed. Similar to the present study, no sex difference were found among 19 horses ridden by inexperienced riders. The present study included a larger sample size (*n* = 492) compared with prior studies evaluating spinal segmental dysfunctions.^3^, ^6^, ^7^ A larger and less homogeneous sample more accurately represents the equine population engaged in athletic activities, thereby supporting more consistent data. Sex did not appear to influence the occurrence of spinal dysfunction.^3^ The number of male and female horses in this study was comparable, though slightly divergent from official data on the sexes of showjumping competitors. According to the *Fédération Équestre Internationale* (www.fei.org) in 2019 (prior to the COVID‐19 pandemic), there were 20,229 mares, 19,103 geldings and 5909 stallions (25,012 males total) registered as jumpers. These figures indicate a higher prevalence of males competing in jumping, in contrast to this study, where sexes were represented more equally.

Age‐related effects were observed specifically in the cervical spine. Osteoarthritic degenerative changes in younger animals are often attributed to malformation issues, affecting the more mobile cervical vertebral segments (C3‐C4‐C5), frequently resulting in dynamic stenotic compression of the spinal cord and subsequent neurological deficits,^8^ potentially leading to withdrawal from athletic careers. The higher prevalence of cervical segmental dysfunction found in older horses (G4) may be explained by an increased prevalence of osteoarthrosis within this study's population. Cervical osteoarthrosis is common, particularly caudal to C5,^9^ and can occur in both young and adult horses. This condition is particularly prevalent in Warmbloods, which have anatomically heavier heads and necks compared with other breeds, rendering them more susceptible.^8^ It remains unclear whether this issue is solely a breed effect, specifically among older Warmbloods with heavier neck and head conformation, or whether age alone contributes to increased tissue degeneration and functional limitations, as previously suggested.^10^

A relationship between the type of equestrian discipline and the types of injuries horses are prone to develop was previously described.^11^, ^12^ Given this context, further studies involving horses of different breeds or disciplines could clarify whether age is a risk factor for the high prevalence of cervical segmental dysfunctions in jumping Warmbloods. In the present study, 59.8% of subjects exhibited segmental dysfunction at C1, despite being ridden by riders of varying technical skills. A previous study reported 83% of subjects with segmental dysfunction at the C1 level,^6^ although it involved a significantly smaller sample size (*n* = 14) of privately owned horses of mixed age and sex. Poor riding technique has been recognised as a contributor to mechanical overload and stress on spinal joints, leading to segmental dysfunction.^3^ The lower prevalence of dysfunctional C1 in the present study may also be attributed to the high skill level of many of the riders involved as it is possible to speculate that better riding technique may minimise undue stress on the horse's structure.

Among the 467 patients where information on clinical signs of muscle pain was noted, 295 (63.2%) exhibited no signs of pain or discomfort, with only one‐fifth showing discomfort at palpation with muscle fasciculation and/or spasm (18.8%) or overt clinical signs of pain (18%). However, due to the lack of specific information regarding the location of muscle pain or discomfort areas in the database, a direct relationship between the location of dysfunctional segments and the presence of pain or altered muscle tone could not be established. More detailed studies may be necessary to elucidate the correlation between spinal segmental dysfunction and muscle alterations in horses. A study involving competition Western horses with back pain identified muscle hypertonicity, although it was not closely linked to the prevalence of muscle pain.^7^ Data on muscle tone were available for 470 out of 492 animals in our study. Almost two‐thirds of the horses (293/470) exhibited alterations such as increased tone (53.6%) or muscle contractures (8.3%), potentially due to the high prevalence of spinal segmental dysfunction combined with athletic demands. Normal muscle tone was observed in 177 horses (37.7%). Overall, 63.2% of horses exhibited muscle hypertonicity, and 36.8% showed signs of muscle pain. These findings could support that veterinary chiropractic is being used not only for managing back pain but also for early detection of spinal dysfunction and optimisation of neuromusculoskeletal health.^1^, ^4^

The present study revealed a higher frequency of spinal dysfunction in the lumbar spine compared with other spinal regions, which aligns with findings from a previous study that reported a similar trend among subjects in Western competitions, primarily affecting the caudal lumbar area.^7^ However, the current study identified the cranial to mid‐lumbar segments (L2–L4) as the most affected. The iliopsoas muscle group, which plays a crucial role in lumbosacral, pelvic, and hip joint flexion, as well as pelvic limb protraction, also exerts considerable force during the jumping take‐off and propulsion phases at the lumbar area. Muscles of the thoracic sling elevate the thorax and forelimbs during the take‐off and landing phases of jumping.^13^

The high prevalence of segmental dysfunction from C3 to T10 found in this study (Table 1) can be attributed to the substantial biomechanical forces transmitted by muscles of the thoracic sling during the thoracic elevation and propulsion phases, as well as load absorption during landing. Therefore, vertebrae in close anatomical relation to these potent muscle groups involved in the jumping phases are subject to significant biomechanical load, associated with a higher prevalence of segmental spinal dysfunction.

The reliability of identifying the precise location of dysfunctional segments through motion palpation may be questionable, influenced by the examiner's skill, experience, and the area being examined. A human study assessed the accuracy of spinal dysfunction diagnosis with two experienced examiners, found that the reliability of determining the level of vertebral dysfunction was higher for the cervical and lumbar regions compared with the thoracic area.^14^ By correlation, a margin of error may exist in veterinary chiropractic regarding the precise listing of dysfunctional segments. Another consideration is the anatomical variation in the number of vertebrae reported by various authors.^15^, ^16^, ^17^, ^18^, ^19^, ^20^ A post‐mortem study involving 38 Thoroughbreds found that only 61% had the standard vertebral formula (C7, T18, L6 and S5).^4^ However, this potential error may be negligible in the present study when considering the spine as a whole (cervical, thoracic or lumbar) and when accounting for the examiner's prior experience.

Regarding the long‐term effects of manipulative therapy, it has been observed that after two chiropractic sessions spaced 3 weeks apart, it persisted for up to 8 months.^21^ Data from animals that received veterinary chiropractic treatment within weeks prior to data collection were excluded from the present study, ensuring a ‘washout’ period, an interval deemed sufficient for the reappearance of segmental dysfunctions in cases of continuous stress factors that may remained unaddressed, such as ill‐fitting saddles, poor riding techniques, uneven terrain, poor conformation or lameness. The quantity of spinal segmental dysfunction may have been lower in this study as animals with lameness have been excluded from the sample. It is known that lameness alters the kinematics of the vertebral column^22^ and can increase the number of dysfunctional segments. However, the sample used in this study is of considerable size, encompassing horses with varied musculoskeletal health, competing at all levels, ridden by riders with varied skill sets and therefore constituting a wide‐ranging and unbiased sample, so providing a realistic overview of the spinal health of equine athletes in jumping training and performance.

Identifying and correcting factors that lead to chronic overload is crucial. Specific rehabilitation and strengthening exercise plans can be prescribed to improve joint stability and prevent the recurrence of segmental spinal dysfunctions, thereby supporting these horses continued athletic performance.

In conclusion, jumping horses are subjected to a high prevalence of segmental dysfunction across all spinal regions, as no horse in this study was entirely free from spinal segmental dysfunctions. The lumbar spine, thoracic sling area and atlanto‐occipital joint are more frequently affected. Additionally, the prevalence of segmental dysfunction in the cervical spine increases with age among athletes participating in the jumping discipline.

## FUNDING INFORMATION

Not applicable.

## CONFLICT OF INTEREST STATEMENT

The authors declare no conflicts of interest.

## AUTHOR CONTRIBUTIONS

**Claudia R. Patricio:** Conceptualization; formal analysis; investigation; methodology; writing – original draft; data curation; resources; writing – review and editing. **Gustavo H. Z. Winter:** Formal analysis; writing – review and editing; methodology; supervision. **Petra Garbade:** Conceptualization; methodology; writing – review and editing; data curation; formal analysis; supervision.

## DATA INTEGRITY STATEMENT

Claudia R. Patricio had full access to all the data in the study and takes responsibility for the integrity of the data and the accuracy of the data analysis.

## ETHICAL ANIMAL RESEARCH

Research ethics committee oversight not required by this journal: retrospective study of clinical records.

## INFORMED CONSENT

Not stated.

## PEER REVIEW

The peer review history for this article is available at https://www.webofscience.com/api/gateway/wos/peer-review/10.1111/evj.14541.

## Data Availability

The data that support the findings of this study are available upon reasonable request from the corresponding author. Open data sharing exemption granted by the editor.

## References

[1] Haussler KK . Back problems. Chiropractic evaluation and management. Vet Clin North Am Equine Pract. 1999;15(1):195–209. 10.1016/s0749-0739(17)30172-4 10218250

[2] Schultz JA , Haffner JC , Wooten MS , Hoffman R , Spooner H . 24 the effect of chiropractic treatment on performance and behavior of lesson horses. J Equine Vet Sci. 2015;35(5):393. 10.1016/j.jevs.2015.03.032

[3] Lesimple C , Fureix C , Menguy H , Hausberger M . Human direct actions may alter animal welfare, a study on horses (*Equus caballus*). PLoS One. 2010;5(4):e10257. 10.1371/journal.pone.0010257 20442766 PMC2860978

[4] Haussler KK . Application of chiropractic principles and techniques to equine practice. Proc Ann Conv AAEP. 1997;43:312–318.

[5] Landman MA , de Blaauw JA , van Weeren PR , Hofland LJ . Field study of the prevalence of lameness in horses with back problems. Vet Rec. 2004;155(6):165–168. 10.1136/vr.155.6.165 15357376

[6] Langstone J , Ellis J , Cunliffe C . A preliminary study of the effect of manual chiropractic treatment on the splenius muscle in horses when measured by surface electromyography. Equine Vet J. 2015;47(S48):18. 10.1111/evj.12486_41 26374981

[7] Haussler KK , Manchon PT , Donnell JR , Frisbie DD . Effects of low‐level laser therapy and chiropractic care on back pain in quarter horses. J Equine Vet Sci. 2020;86:102891. 10.1016/j.jevs.2019.102891 32067657

[8] Zsoldos RR , Groesel M , Kotschwar A , Kotschwar AB , Licka T , Peham C . A preliminary modelling study on the equine cervical spine with inverse kinematics at walk. Equine Vet J. 2010;42(S38):516–522. 10.1111/j.2042-3306.2010.00265.x 21059054

[9] Clayton HM , Kaiser LJ , Lavagnino M , Stubbs NC . Dynamic mobilisations in cervical flexion: effects on intervertebral angulations. Equine Vet J. 2010;42(S38):688–694. 10.1111/j.2042-3306.2010.00196.x 21059082

[10] Maldonado MD , Parkinson SD , Story MR , Haussler KK . The effect of chiropractic treatment on limb lameness and concurrent axial skeleton pain and dysfunction in horses. Animals (Basel). 2022;12:2845. 10.3390/ani12202845 36290230 PMC9597761

[11] Fonseca BPA , Alves ALG , Nicoletti JLM , Thomassian A , Hussni CA , Mikail S . Thermography and ultrasonography in back pain diagnosis of equine athletes. J Equine Vet Sci. 2006;26:507–516. 10.1016/j.jevs.2006.09.007

[12] Jeffcott LB . Disorders of the thoracolumbar spine of the horse—a survey of 443 cases. Equine Vet J. 1980;12:197–210. 10.1111/j.2042-3306.1980.tb03427.x 7439145

[13] Denoix JM . Spinal biomechanics and functional anatomy. Vet Clin North Am Equine Pract. 1999;15:27–60. 10.1016/S0749-0739(17)30162-1 10218240

[14] Cooperstein R , Young M , Haneline M . Interexaminer reliability of cervical motion palpation using continuous measures and rater confidence levels. J Can Chiropr Assoc. 2013;57(2):156–164. 23754861 PMC3661183

[15] Rooney JR . Congenital equine scoliosis and lordosis. Clin Orthop Relat Res. 1969;62:25–30. 5813189

[16] Jeffcott LB . Radiographic features of the normal equine thoracolumbar spine. Vet Radiol. 1979;20:140–147. 10.1111/j.1740-8261.1979.tb01192.x

[17] Sisson S . Articulações dos equinos. In: Getty R , editor. Anatomia dos animais domésticos. 5a ed. Rio de Janeiro, RJ, Brazil: Guanabara Koogan; 1986. p. 324–349.

[18] Townsend HG , Leach DH , Fretz PB . Kinematics of the equine thoracolumbar spine. Equine Vet J. 1983;15(2):117–122. 10.1111/j.2042-3306.1983.tb01732.x 6873044

[19] Haussler KK , Stover SM , Willits NH . Developmental variation in lumbosacropelvic anatomy of thoroughbred racehorses. Am J Vet Res. 1997;58(10):1083–1091. 9328659

[20] Haussler KK . Anatomy of the thoracolumbar vertebral region. Vet Clin North Am Equine Pract. 1999;15(1):13–26. 10.1016/s0749-0739(17)30161-x 10218239

[21] Faber MJ , van Weeren PR , Schepers M , Barneveld A . Long‐term follow‐up of manipulative treatment in a horse with back problems. J Vet Med A Physiol Pathol Clin Med. 2003;50(5):241–245. 10.1046/j.1439-0442.2003.00527.x 14567510

[22] Gomez Alvarez CB , L'ami JJ , Moffat D , Back W , van Weeren PR . Effect of chiropractic manipulations on the kinematics of back and limbs in horses with clinically diagnosed back problems. Equine Vet J. 2008;40:153–159. 10.2746/042516408X250292 18089466

